# Identification and experimental validation of biomarkers associated with the endocannabinoid system in major depressive disorder

**DOI:** 10.1186/s41065-025-00558-6

**Published:** 2025-09-26

**Authors:** Linlin Wang, Min Chen, Xujuan Li, Yufeng Li

**Affiliations:** 1 Shulan (Hangzhou) Hospital, Shulan International Medical College, Zhejiang Shuren University, Hangzhou, 310022 P. R. China; 2 Neurology Department, Qingdao Hospital,University of Health and Rehabilitation Sciences(Qingdao Municipal Hospital), No.1 Jiaozhou Road, Shibei District, Qingdao, 266033 China; 3 Department of Psychological Clinic, Qingdao Hospital, University of Health and Rehabilitation Sciences(Qingdao Municipal Hospital), No.1 Jiaozhou Road, Shibei District, Qingdao, 266033 China

**Keywords:** Major depressive disorder, Endocannabinoid system, Biomarkers, Nomogram

## Abstract

**Background:**

The endocannabinoid system (ES) plays a pivotal role in modulating central nervous system activity in response to emotional stimuli. This study aimed to identify and validate biomarkers associated with ES-related genes (ES-RGs) in major depressive disorder (MDD), providing insights into potential therapeutic targets.

**Methods:**

Datasets GSE52790 and GSE38206 were analyzed in this study. Overlapping differential expression analysis and weighted gene co-expression network analysis (WGCNA) were integrated to identify intersecting genes. Candidate genes were selected through protein-protein interaction (PPI) analysis. Biomarker identification involved the integration of machine learning techniques, gene expression data, and receiver operating characteristic (ROC) analysis. A nomogram was developed and evaluated using these biomarkers as key indicators. Comprehensive analyses, including functional exploration, immune infiltration assessment, regulatory network construction, and reverse transcription-quantitative polymerase chain reaction (RT-qPCR) validation, were conducted.

**Results:**

Mitochondrial ribosome protein S11 (MRPS11) and mitochondrial serine hydroxymethyltransferase2 (SHMT2) were identified as significant biomarkers for MDD, with markedly reduced expression in patient samples. These findings were validated by RT-qPCR analysis. The development of a biomarker-based nomogram successfully predicted MDD risk. Enrichment analysis highlighted the co-enrichment of both biomarkers in the “ribosome” pathway. Differential immune cell analysis revealed four immune cell types distinguishing MDD from control samples. Moreover, five key miRNAs targeting these biomarkers were predicted, along with 31 lncRNAs targeting the miRNAs, establishing an lncRNA-miRNA-mRNA network. Ten transcription factors (TFs) targeting the biomarkers were also identified, leading to the construction of a TF-mRNA network. Furthermore, 15 drugs targeting MRPS11 and 56 drugs targeting SHMT2 were identified, resulting in the formation of a biomarker-drug network. These findings may inform more precise and personalized therapeutic strategies for MDD.

**Conclusion:**

MRPS11 and SHMT2 were identified as biomarkers for MDD through the validation of their expression patterns in clinical samples. This study provides a theoretical foundation for the development of targeted therapies for MDD.

**Supplementary Information:**

The online version contains supplementary material available at 10.1186/s41065-025-00558-6.

## Introduction

Major depressive disorder (MDD) is one of the most prevalent and debilitating mental health conditions worldwide, characterized by persistent sadness, anhedonia, fatigue, reduced appetite, and disrupted sleep, often accompanied by suicidal ideation, leading to significant impairment in daily activities and psychosocial functioning [1]. The World Health Organization (WHO) estimates that more than 264 million people globally are affected by depression [2]. In 2018, MDD ranked third in the WHO’s global disease burden assessment, with projections indicating it will become the leading cause of disease burden by 2030 [3]. Despite extensive research, the etiological pathways of MDD remain complex and not fully understood. Similarly, the mechanisms underlying the effectiveness of pharmacological treatments for MDD remain unclear [4]. Therefore, identifying biomarkers for MDD could significantly aid in the diagnosis and treatment of patients.

The endocannabinoid system (ES), first discovered in relation to the Cannabis plant, plays a vital role in regulating and maintaining human health [5]. This system is comprised of lipid-based retrograde neurotransmitters, known as endocannabinoids, which bind to the cannabinoid CB1 and CB2 receptors, triggering a range of physiological and molecular responses. These receptors are widely distributed throughout the human body, including in immune cells, the brain, connective tissues, organs, and various glands [5]. Activation of these receptors inhibits neurotransmitter release [6]. This regulatory mechanism is essential for various brain functions, including emotional, motor, and cognitive processes, while contributing to the overall maintenance of brain homeostasis. The ES includes receptors, their endogenous lipid ligands (endocannabinoids), and the enzymes responsible for their biosynthesis and catabolism [7]. These enzymes regulate numerous physiological processes, including those related to anxiety and depression [8, 9]. The two most famous endocannabinoids are arachidonic acid derivatives, namely arachidonoyl ethanolamide, and 2-arachidonoyl glycerol (2-AG) [10].

The pathophysiology of MDD has been linked to the ES [11]. Previous studies have identified alterations in 2-AG, anandamide, and CB1 receptors in the brains of individuals with unipolar depression. Dysregulation of the ES has been associated with antidepressant use, disease stage, and symptom severity in depression [12].

Exploring the relationship between the ES and MDD may enhance our understanding of the disorder’s pathogenesis and provide novel insights for therapeutic strategies. Utilizing data from the GEO database, this study employed various methodologies, such as differential expression analysis, machine learning algorithms, protein-protein interaction (PPI) analysis, weighted gene co-expression network analysis (WGCNA), and receiver operating characteristic (ROC) analysis, to identify biomarkers associated with the ES in MDD. The presence of these biomarkers in clinical samples was confirmed *via reverse transcription-quantitative polymerase chain reaction* (RT-qPCR) analysis. Additionally, functional exploration, nomogram model development, immune infiltration analyses, regulatory network construction, and drug prediction analyses were performed to further investigate the underlying mechanisms of these biomarkers. These comprehensive analyses offer valuable insights into the diagnosis and treatment of MDD.

## Materials and methods

### Data collection

Transcriptome data for MDD were retrieved from the GEO database (https://www.ncbi.nlm.nih.gov/) [13]. The GSE52790 dataset (platform: GPL17976) included 10 MDD and 12 control peripheral blood samples, while the GSE38206 dataset (platform: GPL13607) contained 18 MDD and 18 control peripheral blood mononuclear cells (PBMCs) samples. Additionally, 10 ES-related genes (ES-RGs) were sourced from the GenBank (www.ncbi.nlm.nih.gov*)* [14] database using the search term “endocannabinoid system” (species: Homo sapiens; time: 2024,07,31.). These genes included DAGLA, HCRTR1, ABHD12, DAGLB, ABHD6, HTR2A, CNR1, ABHD4, HTR1A, and GPR55.

### Differential expression analysis

Differentially expressed genes (DEGs) between MDD and control samples in the GSE52790 dataset were identified using the “limma” package (version 3.58.1) [15] (adjusted *P* < 0.05). The DEGs were visualized using volcano plots and heat maps. Volcano plots were generated using the “ggplot2” package (v 3.4.4) [16], while heat maps were produced using “ComplexHeatmap” (v 2.16.0) [17].

### WGCNA

To assess the expression variation of ES-RGs between MDD and control samples, the Wilcoxon test [18]was applied. Significant ES-RGs (*P* < 0.05) were used to calculate the ES-RGs score using the single-sample gene set enrichment analysis (ssGSEA) from the “GSVA” package (v 1.49.4) [19]. WGCNA was then performed to identify the key module most strongly associated with the ES-RGs score using the “WGCNA” package (v 1.72-5) [20]. Initial clustering of all samples excluded outliers, and an optimal soft threshold was determined to achieve a scale-free topology with an R^2^ value greater than 0.800, ensuring mean connectivity remained close to zero. A co-expression matrix was constructed with a minimum module size of 100 genes, and the module dendrogram was cut using a MEDissThres value of 0.300, generating distinct gene modules, each represented by a unique color. Pearson correlation coefficients between the ES-RGs score and each gene module were computed (|cor| >0.300, *P* < 0.050). Modules showing the strongest correlation with the ES-RGs score were designated as key modules. Gene significance (GS) and module membership (MM) analyses were performed, and genes within key modules with GS > 0.600 and MM > 0.500 [21]were identified as key module genes.

### Identification of candidate genes

Intersection genes were identified by overlapping DEGs and key module genes using the “VennDiagram” package (version 1.7.3) [22]. Gene Ontology (GO) analysis was performed to explore the biological roles of these intersection genes using the “clusterProfiler” package (v 4.10.0) [23] (*P* < 0.050). Additionally, REACTOME and Kyoto Encyclopedia of Genes and Genomes (KEGG) pathway analyses were conducted to investigate the pathways associated with the intersection genes (*P* < 0.050). To examine protein interactions among the intersection genes, a PPI network was constructed using the STRING database [24] (score > 0.400) [25] and visualized with Cytoscape (v 3.10.0) [26]. The maximum neighborhood component (MNC) algorithm [27] was used to identify candidate genes, with the top 10 genes ranked by the MNC algorithm selected for further analysis.

### Determination of biomarkers

The candidate genes were subsequently subjected to least absolute shrinkage and selection operator (LASSO) and support vector machine recursive feature elimination (SVM-RFE) to identify feature genes. Specifically, LASSO was performed using the “glmnet” package (v 4.1-8) [28] with 10-fold cross-validation, selecting the minimum lambda value and non-zero coefficients. For SVM-RFE, the “e1071” package (v 1.7–13) [29] was employed. The overlap of genes identified by both LASSO and SVM-RFE was then designated as feature genes. Gene expression analyses of these feature genes were conducted in the GSE52790 and GSE38206 datasets. Genes that exhibited significant differential expression (*P* < 0.050) and displayed consistent expression patterns across both datasets were defined as candidate biomarkers for further analysis. ROC curves for the candidate biomarkers were generated using the “pROC” package (v 1.18.4) [30] in both datasets, and the area under the curve (AUC) was calculated. Biomarkers with an AUC greater than 0.700 were considered significant.

### Construction and validation of nomogram

Based on the identified biomarkers, a nomogram was constructed using the “rms” package (v 6.7-0) [31]. To evaluate the nomogram’s accuracy, calibration curves and decision curves were plotted. Additionally, the ROC curve was generated using “pROC“(1.18.4) [32] to assess the model’s performance.

### Functional analysis of biomarkers

To explore the genes functionally associated with the biomarkers, a gene-gene interaction (GGI) network was constructed using GeneMANIA (https://genemania.org/) [33]. Furthermore, to investigate the biological roles associated with the biomarkers, Spearman correlations were calculated and ranked between the identified biomarkers and other genes in the GSE52790 dataset. The ‘c2.cp.kegg.v7.5.1.symbols.gmt’ gene set was downloaded from the Molecular Signatures Database (MSigDB) (https://www.gsea-msigdb.org/gsea/msigdb/index.jsp) [34] as the background set, and gene set enrichment analysis (GSEA) was performed (q < 0.05).

### Immune infiltration analysis

In the GSE52790 dataset, the ssGSEA method from the “GSVA” package (1.49.4) [35] was applied to calculate scores for 28 different immune cell types [36] across all samples. Cells exhibiting significant differential infiltration between MDD and control samples (Wilcoxon test, *P* < 0.050) were selected. Spearman correlation analysis was then performed to explore the relationships between differential immune cells and the identified biomarkers (|cor| >0.300, *P* < 0.050).

### Subcellular and chromosomal localization analyses

The subcellular localization of biomarkers was explored to better understand their biological functions. The mRNALocater database (http://bio-bigdata.cn/mRNALocater) [37] was used to determine the subcellular distribution of biomarkers. Additionally, the chromosomal locations of the biomarkers were visualized using “RCircos” (v 1.2.2) [38].

### Regulation network analysis

The DIANA-microT and MicroCosm databases from the “multiMiR” package (version 1.14) [39] were used to identify miRNAs targeting the biomarkers. Key miRNAs were selected by overlapping the predictions from both databases. The StarBase (https://starbase.sysu.edu.cn/index.php) [40] was then employed to identify lncRNAs targeting these key miRNAs. These relationships were organized into an lncRNA-miRNA-mRNA network, which was visualized using Cytoscape. Furthermore, transcription factors (TFs) targeting the biomarkers were predicted using the Position Weight Matrix Analysis for Transcription Factor Binding Sites Database (PASTAA, https://trap.molgen.mpg.de/PASTAA.htm) [41], and a TF-mRNA network was constructed. To explore therapeutic drugs linked to the biomarkers, the DsigDB (https://dsigdb.tanlab.org/DSigDBv1.0/) [42] was utilized, and a biomarkers-drugs network was created using Cytoscape.

### RT-qPCR

To validate the expression patterns of the biomarkers, RT-qPCR was performed. The blood sample of ten participants (five healthy controls and five cases) [43] were recruited from Shulan (Hangzhou) Hospital, with informed consent obtained from all participants. The study was approved by the Shulan (Hangzhou) Hospital ethics committee (approval number: KY2025049).

Ten freshly collected blood samples were added to a 15 ml centrifuge tube with an equal volume of PBMC separation solution (3 ml for blood < 3 ml). Mixed whole blood was slowly added and centrifuged at 2000 g for 20 min. The liquid was divided into four layers, with PBMCs present in the second circular milky white layer. Carefully aspirate the PBMC layer into a new centrifuge tube, add PBS to 15 ml, and resuspend the cells. Centrifuge 1000 g for 10 min, carefully discard the supernatant, add 1 ml TRIZol and let it stand at room temperature for 10 min to resuspend and lyse the cells (or freeze at -80℃ in a refrigerator). Add 300 ml of chloroform, shake vigorously for 30 s, let it stand at room temperature for 10 min to allow the liquid to separate into layers. Centrifuge at 12,000 g and 4 ℃ for 15 min, and the liquid can be seen to be divided into three layers [44, 45] RNA remains in the colorless upper aqueous phase. Carefully transfer the upper aqueous phase into another EP tube, being careful not to suck into the middle and lower layers (if accidentally sucked, it must be gently squeezed out). Then add an equal volume of ice isopropanol, invert and mix well, let it stand for 10 min (when the sample size is small, it can be placed in a -20 ° C refrigerator overnight to improve RNA extraction rate). After centrifugation at 12,000 g and 4 ° C for 10 min, white RNA precipitate can be seen at the bottom of the tube (when the sample size is small, the precipitate is not visible to the naked eye and does not affect normal operation). Gently tilt the tube opening to discard the supernatant, being careful not to pour out the sediment. Use absorbent paper to dry the tube opening, add 1 ml of 75% ethanol to the sediment, invert a few times to make the sediment float, let it stand for 2 min, centrifuge at 7500 g and 4 ℃ for 5 min, and make the sediment adhere to the bottom of the tube again. Repeat this step twice. Discard the supernatant, invert the centrifuge tube onto absorbent paper, and use a 10ul pipette tip to carefully remove the remaining liquid. Be careful not to remove the precipitate. Let it dry naturally for 20 min or place it on an ultra clean workbench to blow dry, allowing ethanol and water to evaporate as much as possible. The RNA precipitate will become transparent, but it should not be too dry, otherwise it will affect the subsequent dissolution of RNA. Add 20-50ul of RNase free water to the dried RNA precipitate, let it stand for 15 min to completely dissolve the RNA, and take 1ul for concentration detection using Nano drop. Record the RNA purity/concentration to calculate the amount of sample for subsequent reverse transcription steps. The remaining RNA is immediately reverse transcribed or frozen in a -80℃ freezer [45]. Subsequently, 1ul of RNA was taken and the concentration of RNA was measured using a NanoPhotometer N50. The synthesis of cDNA was accomplished through reverse transcription applying the SureScript-First-strand-cDNA-synthesis-kit, with the reverse transcription process being conducted on an S1000TM Thermal Cycler (Bio-Rad, USA). Specifically, remove the components of the reverse transcription kit, melt them at room temperature, centrifuge briefly, place them on ice, and add each reagent and solution in sequence on the ice [46] (Additional file 2). After a brief centrifugation, reverse transcription was performed on a regular PCR machine according to the conditions of Additional file 3. First, dilute the reverse transcription product cDNA with ddH2O (RNase/DNase free) by 5.000–20.000 times [47]. Perform qPCR reaction according to Additional File 4. When performing the sample loading operation, the nozzle should be checked for any air bubbles after each liquid suction. After blowing the liquid into the hole, the nozzle should also be checked for any liquid residue. If there is any residue, the residual liquid instrument should be blown into the corresponding hole, and the sample loading operation must be carried out in one breath. Other experimental operations or unrelated activities should not be carried out at the same time to ensure the consistency of the repeated holes. After a brief centrifugation, perform 40.000 cycles of reaction on a CFX96 real-time quantitative fluorescence PCR instrument under the following conditions, prepare amplification and dissolution curves, and read Ct values [48]. Additional file 5. provides amplification conditions. The sequences of all primers were provided in Additional file 6. In order to perform ROC validation on key genes in clinical samples, ROC curves for the candidate biomarkers were generated using the “pROC” package (v 1.18.4) [30], and the area under the curve (AUC) was calculated. Biomarkers with an AUC greater than 0.700 were considered significant.

### Statistical analysis

Statistical analysis was performed using R (v 4.3.3) [49]. Differences between the two groups were analyzed with the Wilcoxon test (*P* < 0.05).

## Results

### DEGs and key module genes were separately identified

**Fig. 1:**
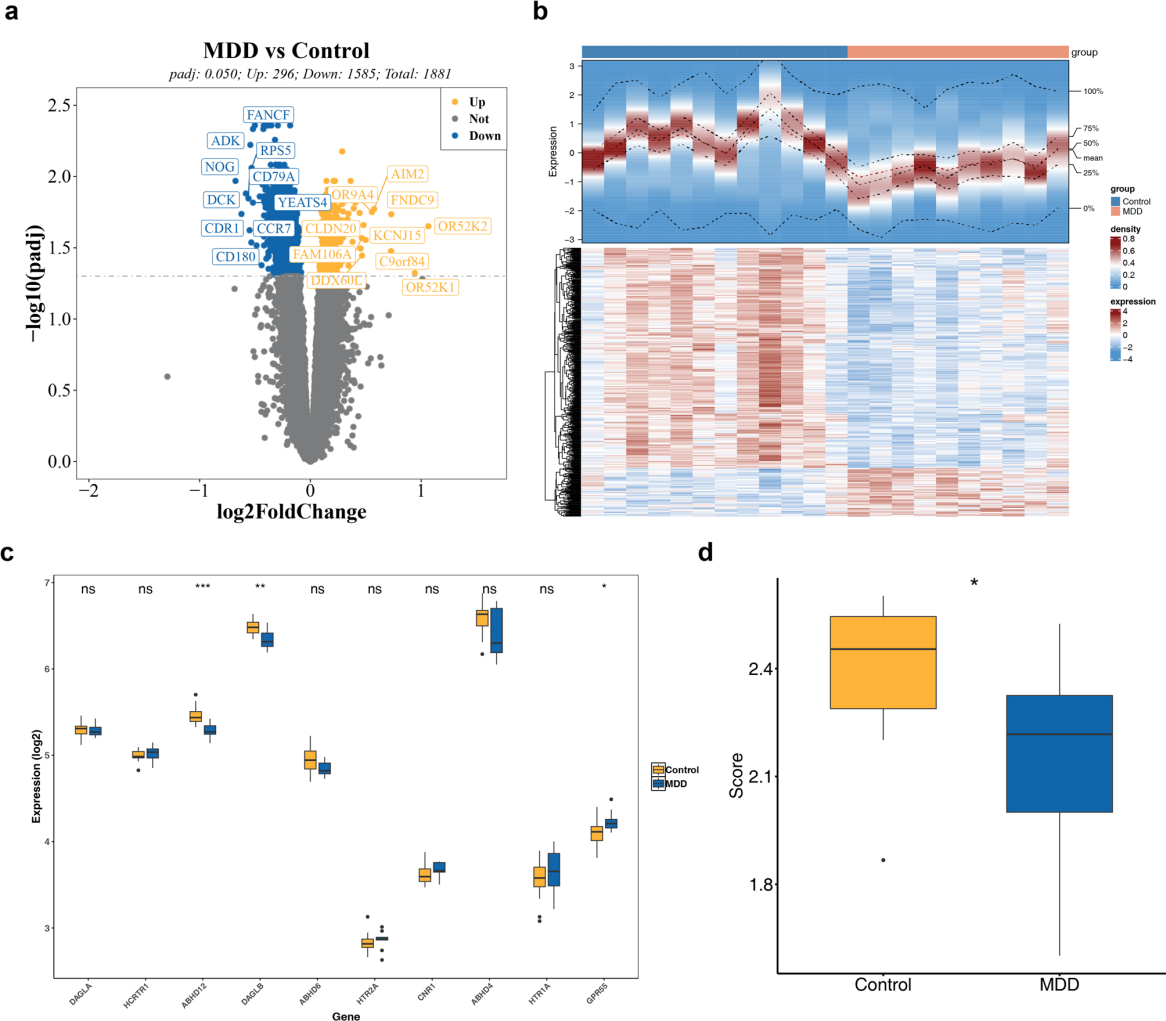
Expression and analysis of differentially expressed genes (DEGs) (**a**) Volcano plot of DEGs1. Each point corresponds to a gene; blue points indicate downregulated genes, yellow points represent upregulated genes, and gray points reflect genes with no significant difference. The dashed line marks the threshold for selecting DEGs. (**b**) Heatmap of DEGs: Each small square represents a gene. Darker colors denote higher expression levels (red for high expression and blue for low expression). (**c**) Box plot of gene expression: Yellow represents normal samples, while blue represents MDD samples. (**d**) Differences in ES-RGs scores between disease and control groups: Blue denotes MDD samples, and yellow represents normal samples. *:*P* < 0.050

A total of 1,881 DEGs were identified, with 296 genes being upregulated and 1,585 genes downregulated in MDD samples (Fig. 1a-b). Among these, three ES-RGs—ABHD12, DAGLB, and GPR55—showed significant expression differences between MDD and control samples (*P* < 0.050) (Fig. 1c). The ES-RGs score, derived from these three genes, was significantly lower in MDD samples (*P* < 0.050) (Fig. 1d).

**Fig. 2:**
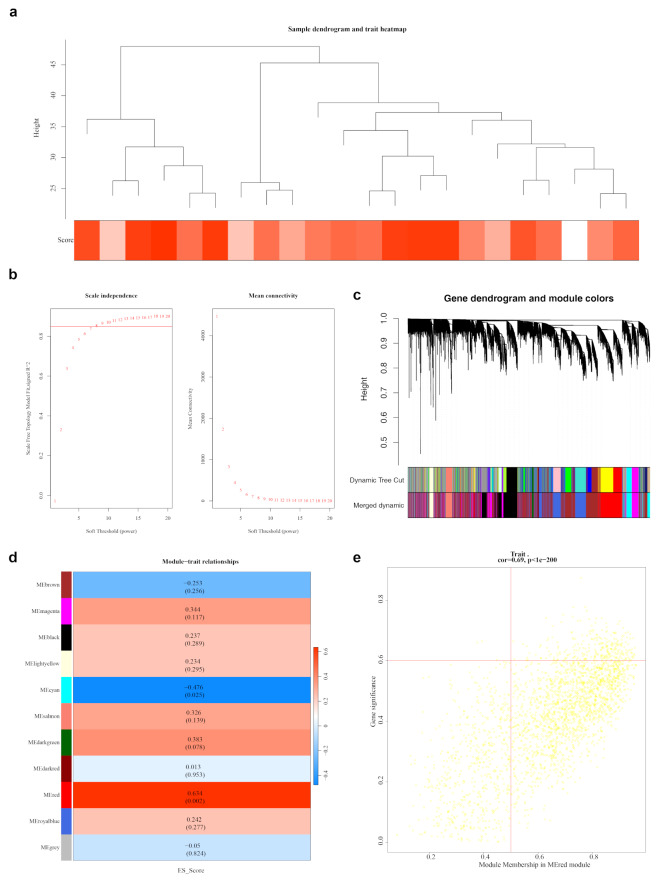
Construction and analysis of gene co-expression networks (**a**) Sample clustering situation: The branches in the figure represent samples, with the vertical axis indicating the height of hierarchical clustering. The clustering of the dataset samples appears to be relatively consistent. (**b**) Scale-free soft threshold distribution. A higher R2 value suggests the network approaches a scale-free distribution. In the right panel, the vertical axis represents the mean adjacency functions of all genes within the corresponding gene module. (**c**) Cluster dendrogram, module cluster tree: Genes are grouped into various modules using hierarchical clustering. Gray represents genes not assigned to any specific module. (**d**) Heatmap of the correlation between modules and ES scores. Each square indicates the correlation coefficient and significance p-value between the module and the ES score. Stronger positive correlations are indicated by red, while stronger negative correlations are indicated by purple. (**e**) Scatter plot of the association strength of the key module GS: The two red solid lines represent the thresholds for MM and GS

In the GSE52790 dataset, no outlier samples were identified or excluded (Fig. 2a). The optimal power value was determined to be 8 (Fig. 2b). A co-expression matrix was generated, resulting in the merging of similar modules, which led to the identification of 11 gene modules (Fig. 2c). The MEred module, consisting of 2,509 genes, exhibited the strongest correlation with the ES-RGs score (cor = 0.630, *P* = 0.002) (Fig. 2d). Applying criteria of |GS| >0.600 and |MM| >0.500, 271 key module genes were identified (Fig. 2e), providing crucial insights into potential genes for MDD.

### Functions of intersection genes were explored, and candidate genes were identified

**Fig. 3:**
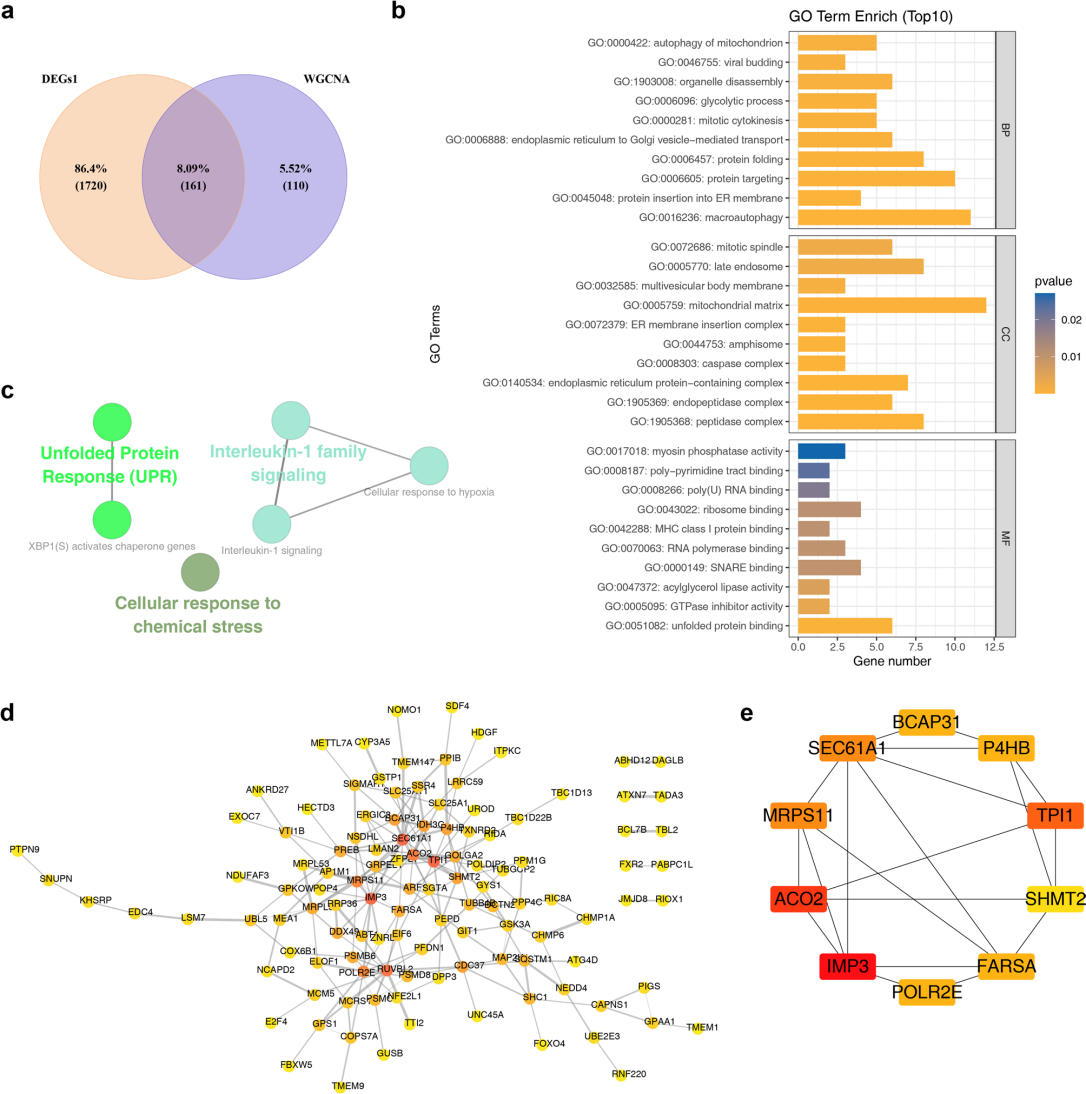
Identification of candidate genes. (**a**) WGCNA-DEGs1 Venn diagram. (**b**) GO enrichment circle plot: The outermost circle represents GO pathway IDs, while the second circle displays the gene count for each pathway. Red denotes upregulated genes, and blue represents downregulated genes. (**c**) KEGG enrichment results. Each node corresponds to a pathway and edges denote the number of shared genes between pathways. Node color indicates the enrichment status classification for each pathway. (**d**) Protein interaction network: Interactions between proteins are represented by lines, with blue nodes representing genes. (**e**) Top 10 genes interaction networks under the MNC algorithm

By intersecting the 1,881 DEGs with the 271 key module genes, 161 intersection genes were identified (Fig. 3a). These genes were significantly associated with 313 GO terms, including 222 biological processes (BPs), 65 cellular components (CCs), and 26 molecular functions (MFs). Notable GO terms included “macroautophagy” (BP), “peptidase complex” (CC), and “unfolded protein binding” (MF) (Fig. 3b). The enriched KEGG pathways included “cellular response to chemical stress,” “interleukin-1 family signaling,” and “interleukin-1 signaling” (Fig. 3c). A PPI network was constructed for the intersection genes, although protein information was available for only 117 genes, resulting in a network comprising 117 nodes and 193 edges (Fig. 3d). For instance, LSM7 was found to interact closely with UBL5 and EDC4. The top 10 genes identified through the MNC algorithm were BCAP31, P4HB, TPI1, SHMT2, FARSA, POLR2E, IMP3, ACO2, MRPS11, and SEC61A1 (Fig. 3e). These genes were recorded as candidate genes for further screening. These results provided valuable targets for in-depth investigation into MDD-related mechanisms.

### Two biomarkers were ascertained

**Fig. 4:**
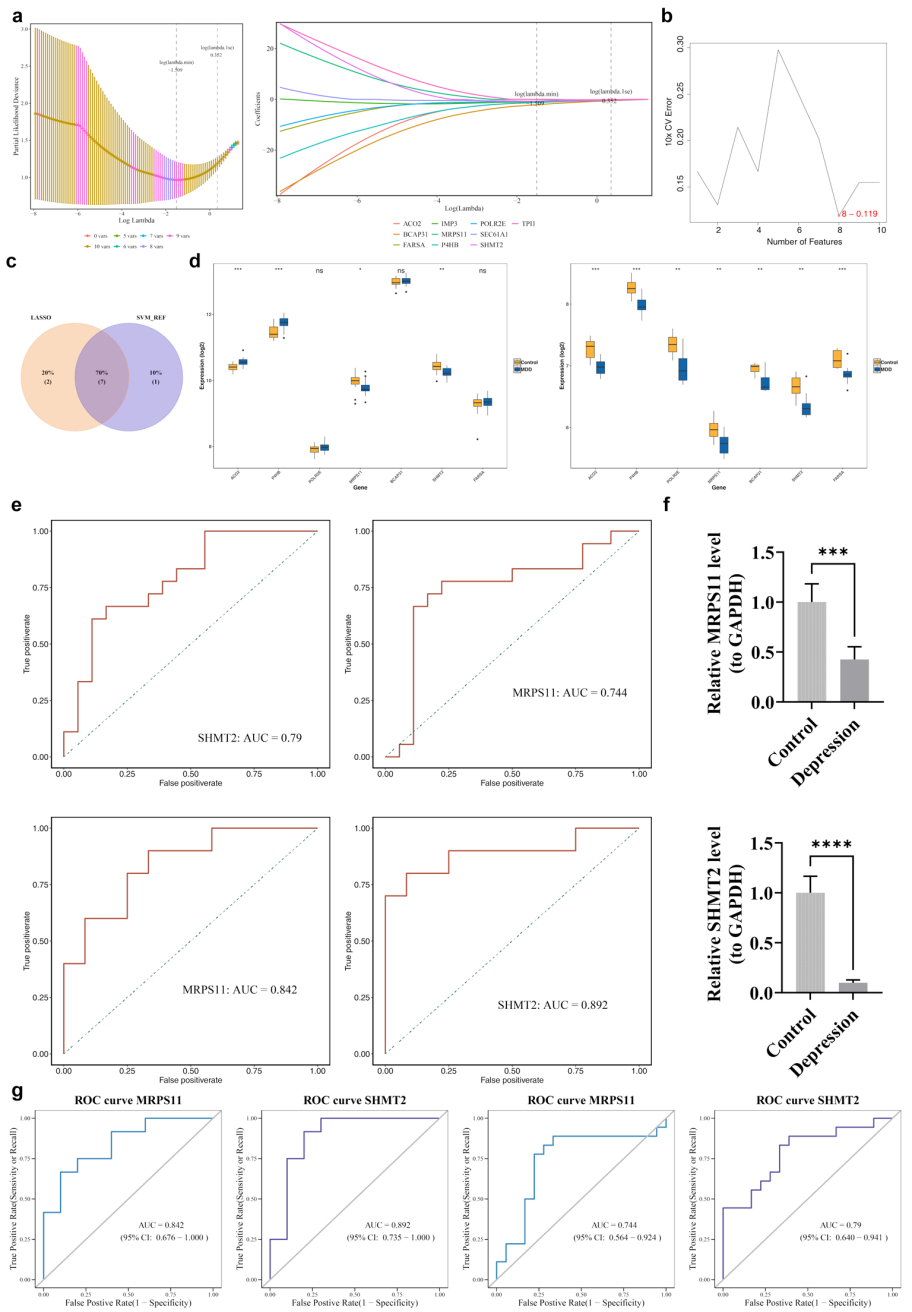
Identification of biomarkers. (**a1**,**2**) Gene selection using LASSO regression. In the first figure, the dashed line on the left indicates the point where the cross-validation error is minimized. (**b**) Relationship between SVM generalization error and the number of features. The x-axis represents the number of feature genes, and the y-axis indicates the generalization error under 10-fold cross-validation. The line’s trend represents the relationship between the number of feature genes and generalization error. (**c**) Intersection of two types of machine learning. (**d1**) Boxplots of the 7 candidate biomarkers in the training set. (**d2**) Boxplots of the 7 candidate biomarkers in the validation set. (**e1**) ROC curves of candidate biomarkers in the training set. (**e2**) ROC curves of candidate biomarkers in the validation set. (**f**) PCR experiment: RT-qPCR analysis showed that the expression patterns of MRPS11 and SHMT2 were significantly lower in MDD samples. ***: *P* < 0.001; ****: *P* < 0.0001

From the 10 candidate genes, LASSO analysis identified nine genes with a log (lambda.min) = -1.509 and non-zero regression coefficients (Fig. 4a). Similarly, eight genes were identified using the SVM-RFE method (Fig. 4b). The seven feature genes were determined by overlapping the gene sets from LASSO and SVM-RFE (Fig. 4c). Notably, MRPS11 and SHMT2 exhibited significantly lower expression in patients with MDD across both the GSE52790 and GSE38206 datasets (Fig. 4d). Both MRPS11 and SHMT2 showed an AUC greater than 0.700 in these datasets, demonstrating strong diagnostic accuracy (Fig. 4e). Therefore, MRPS11 and SHMT2 were identified as biomarkers for MDD. RT-qPCR validation confirmed that the expression of MRPS11 and SHMT2 was significantly lower in MDD samples (Fig. 4f). Cross validation of clinical samples shows MRPS11 and SHMT2 showed an AUC greater than 0.700 in these datasets, demonstrating strong diagnostic accuracy(Fig. 4g). These findings emphasize MRPS11 and SHMT2 as promising biomarkers for further investigation in subsequent analyses.

### A good nomogram was constructed

**Fig. 5:**
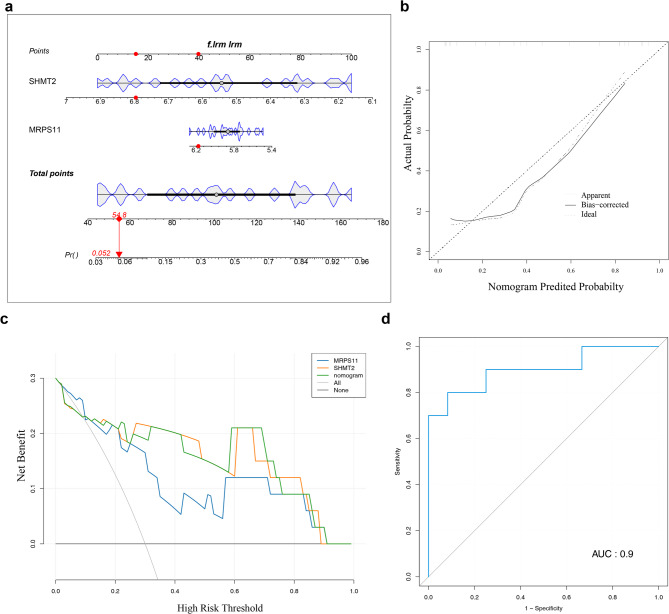
Construction and validation of the nomogram. (**a**) Nomogram of Biomarkers. The figure on the left shows the biomarkers, with the right side displaying scales corresponding to each biomarker, indicating the range of possible values. The “point” represents the individual score associated with each biomarker at different values, while “Total Point” indicates the sum of all individual scores. “Pr” denotes the risk of having MDD. (**b**) Calibration curve of the nomogram. The Ideal line shows the scenario where model predictions perfectly align with actual outcomes. (**c**) DCA curve: The x-axis represents threshold probability, where Pi denotes the probability of patient i being diagnosed with the disease. When Pi exceeds a certain threshold (Pt), the diagnosis is considered positive, and treatment is administered. The y-axis represents the net benefit (NB), the difference between advantages and disadvantages. The various curves represent different clinical diagnostic models, with two lines indicating extreme scenarios. (**d**) ROC curve for nomogram: The x-axis represents specificity, and the y-axis represents sensitivity

A nomogram was constructed based on the two biomarkers, MRPS11 and SHMT2 (Fig. 5a). The nomogram demonstrated that higher total scores were associated with an increased risk of MDD, with accurate model predictions (Fig. 5b). Furthermore, the decision curve analysis (DCA) indicated that the model provided superior net benefit compared to others (Fig. 5c). The model achieved an AUC of 0.900, confirming its excellent predictive performance (Fig. 5d). These results underscore the robust predictive capability of the nomogram for MDD.

### The functions of biomarkers were explored

**Fig. 6:**
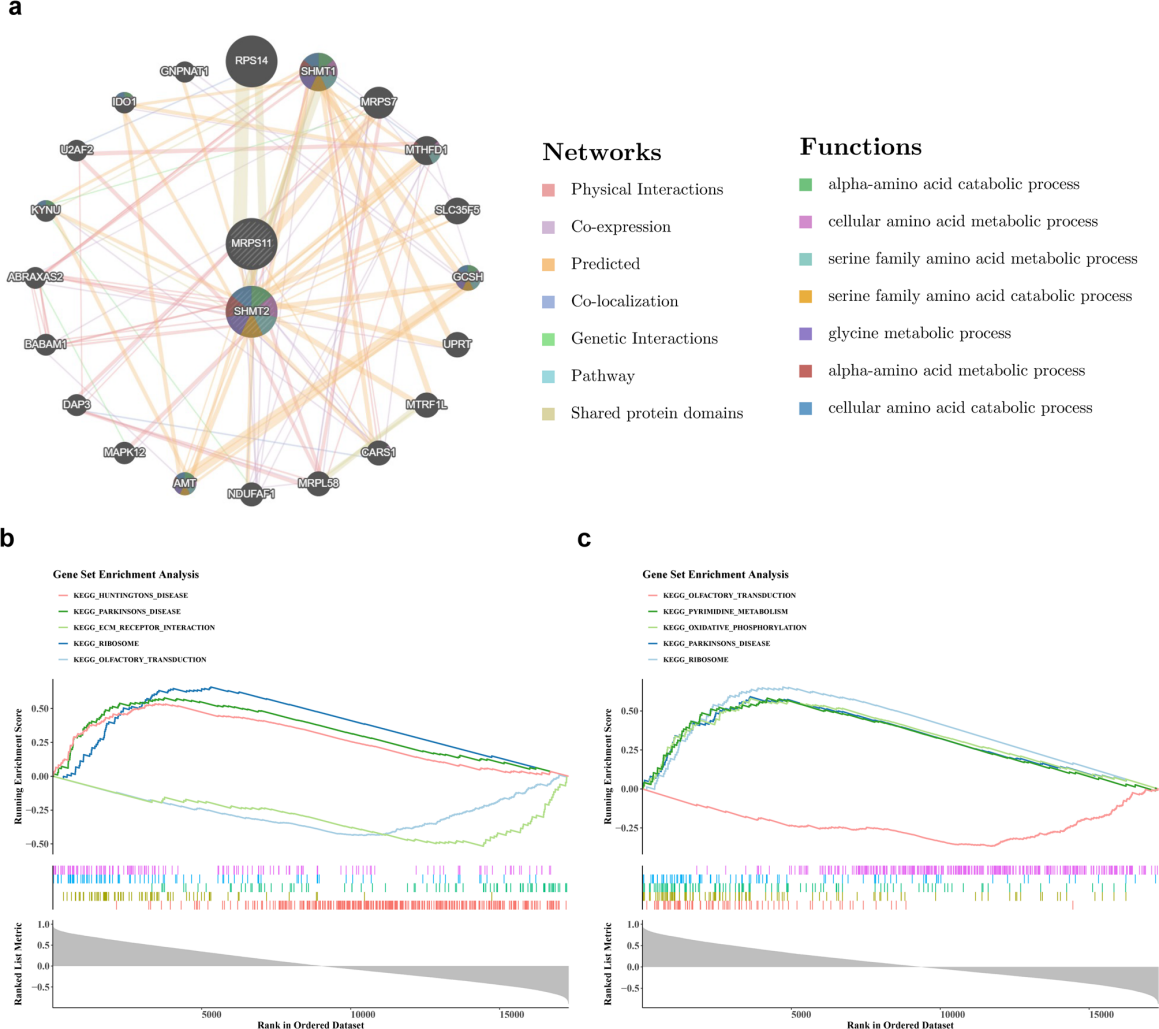
Gene set enrichment analysis. (**a**) GeneMANIA Enrichment Map. (**b**) Gene Enrichment Analysis of GSEAKEGG for MRPS11 (Top 5). (**c**) Gene SHMT2 GSEAGO Enrichment Analysis (Top 5). The figure is divided into three parts. The uppermost section shows the ES value curve, with the highest/lowest point corresponding to the ES value of the pathway. A positive ES value indicates that genes positively correlated with the target gene are dominant in these pathways. The middle section shows the positions of genes in the pathway within the ranked list, with each vertical line representing a gene

Using GeneMANIA, 20 genes associated with the biomarkers were identified. Notable relationships were found, such as the connection between MRPS11 and MRP, and between SHMT2 and Glycine Cleavage System Protein H (GCSH). These relationships are linked to processes such as ‘alpha-amino acid catabolic process’ and ‘cellular amino acid metabolic process’ (Fig. 6a). GSEA revealed significant co-enrichment of MRPS11 and SHMT2 in pathways related to the “ribosome,” “Parkinson’s disease,” and “olfactory transduction” (Fig. 6b-c). These results suggest that the biomarkers are involved in multiple pathways relevant to the pathology of MDD, highlighting their potential as therapeutic targets.

### The immune infiltration differences between MDD and control samples were explored

**Fig. 7:**
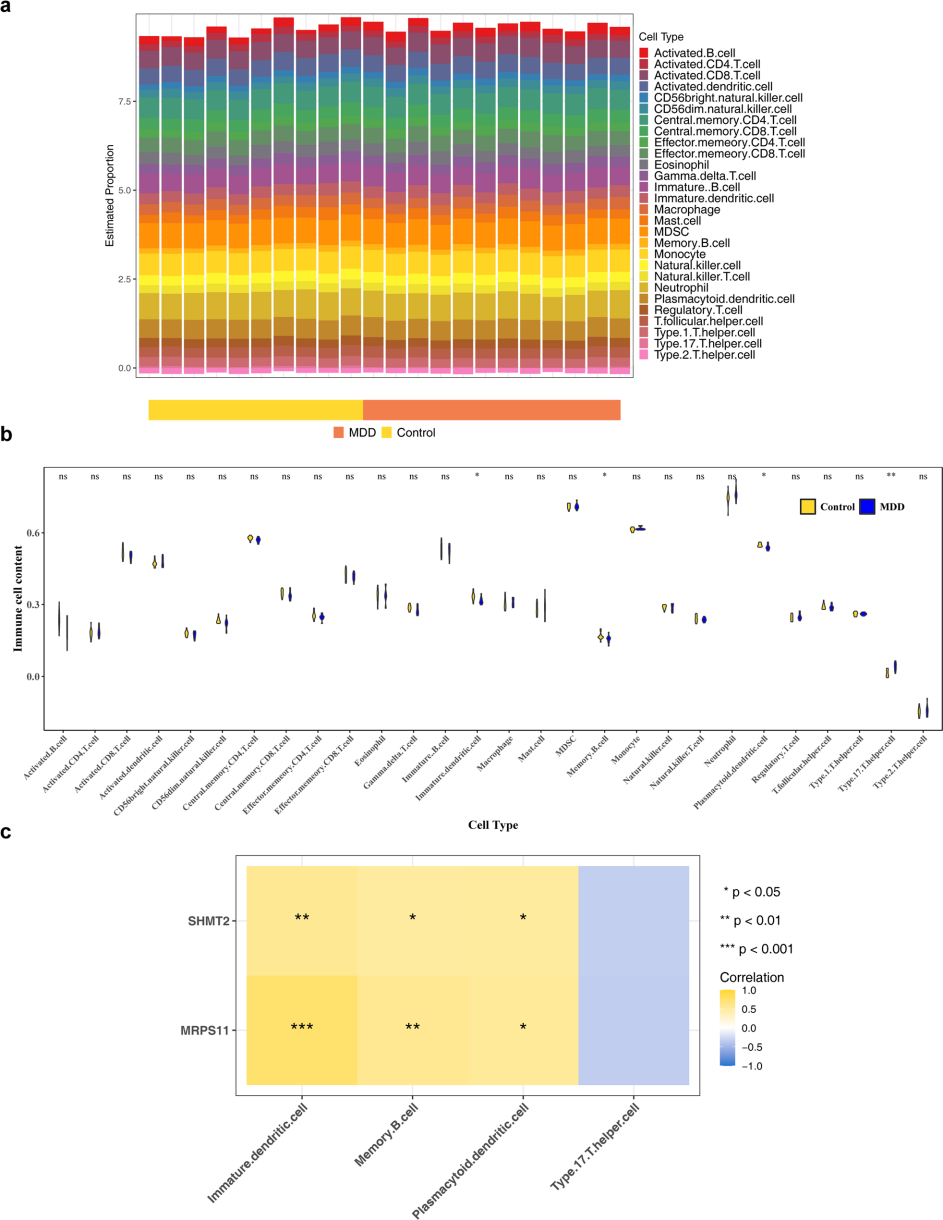
Immunoinfiltration analysis. (**a**) Stacked bar chart of immune cell infiltration proportions. (**b**) Boxplots of immune cells with significant differences. .ns: *P* > 0.050; *: *P* < 0.050; **: *P* < 0.010. (**c**) Differential immune cell correlations: Blue indicates positive correlation, while red indicates negative correlation

Immune infiltration proportions in the GSE52790 dataset are presented in Fig. 7a. Four immune cell types showed significantly different infiltration rates between MDD and control samples (*P* < 0.050). Specifically, immature dendritic cells, memory B cells, and plasmacytoid dendritic cells were more prevalent in controls, while Type 17 T helper cells exhibited higher infiltration in MDD samples (Fig. 7b). Immature dendritic cells displayed the strongest positive correlation with MRPS11 (cor = 0.780, *P* < 0.050) and SHMT2 (cor = 0.563, *P* < 0.050) (Fig. 7c; Table 1). These results suggest that immune cell types and their correlations with the biomarkers play a critical role in MDD, offering valuable insights for the development of targeted immunotherapies for the disorder.

**Table 1:** Correlation analysis between immune cell types and biomarkers

Gene	Immune_cells	Cor	*p*.value
MRPS11	Immature.dendritic.cell	0.748	0.00006
MRPS11	Memory.B.cell	0.574	0.005
MRPS11	Plasmacytoid.dendritic.cell	0.530	0.011
MRPS11	Type.17.T.helper.cell	-0.340	0.121
SHMT2	Immature.dendritic.cell	0.562	0.006
SHMT2	Memory.B.cell	0.497	0.018
SHMT2	Plasmacytoid.dendritic.cell	0.505	0.016
SHMT2	Type.17.T.helper.cell	-0.329	0.135

### The localization of biomarkers was explored

**Fig. 8:**
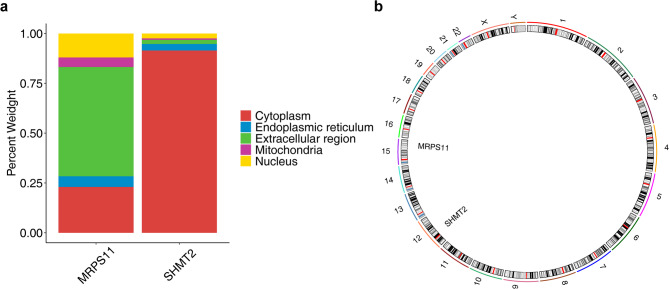
Subcellular localization and chromosomal localization analysis of biomarkers. (**a**) Subcellular localization. (**b**) Gene mapping

Subcellular localization analysis revealed that the proteins translated by MRPS11 are mainly located in the extracellular region, while the proteins translated by SHMT2 are mainly located in the cytoplasm (Fig. 8a). Additionally, SHMT2 was mapped to chromosome 12, whereas MRPS11 was situated on chromosome 15 (Fig. 8b). These findings offer valuable insights into the genomic and cellular contexts of the biomarkers, enhancing our understanding of their functional roles.

### The regulated network was helpful for exploring the potential mechanism for MDD

**Fig. 9:**
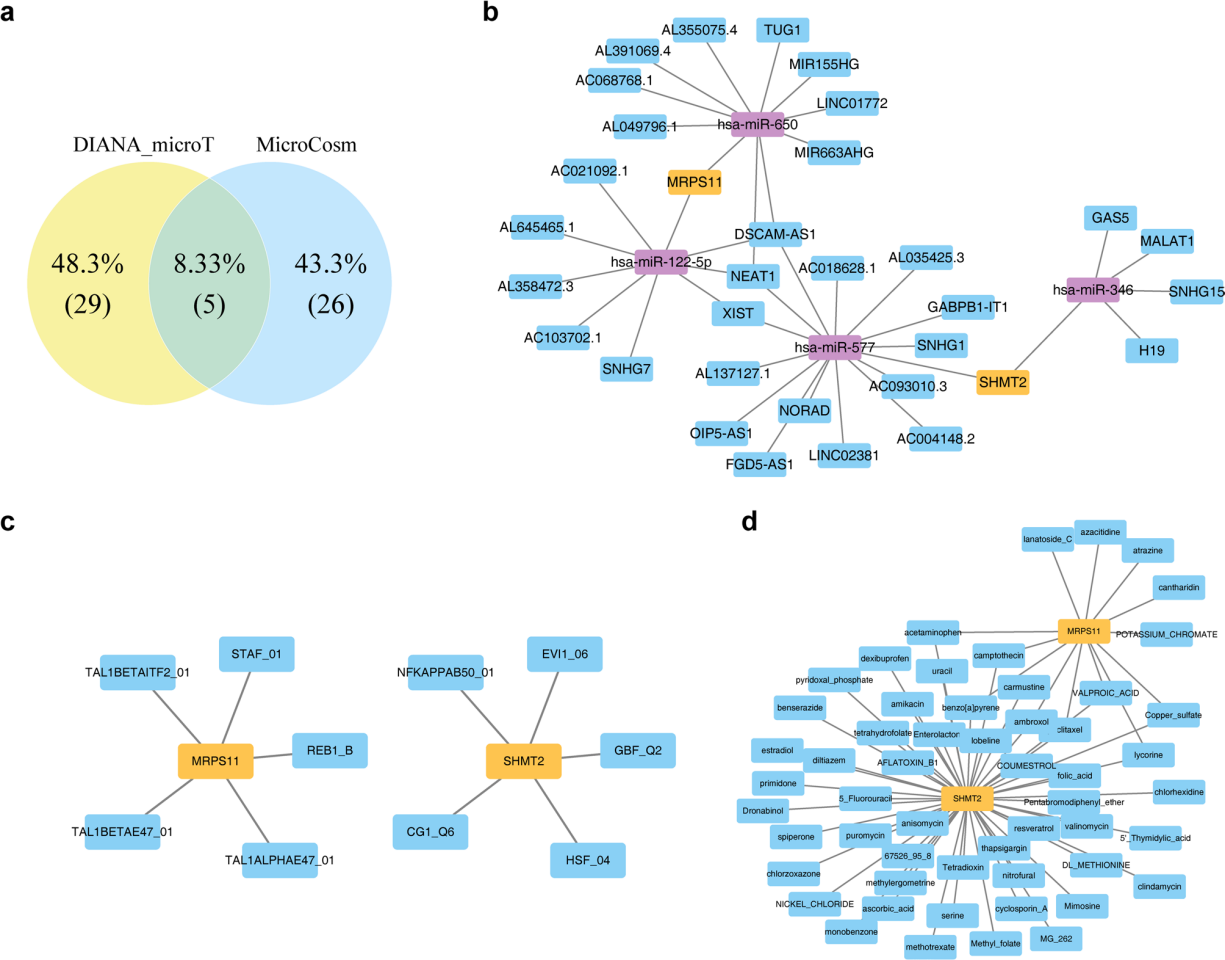
Overview of the molecular regulatory networks and interactions. (**a**) miRNA Venn diagram. (**b**) Molecular regulatory network diagram: Yellow represents genes, purple represents miRNAs, and blue represents lncRNAs. (**c**) TF-mRNA network diagram: Yellow represents genes, and blue represents TFs. (**d**) Gene-drug interaction network diagram

Through querying the DIANA microT and MicroCosm databases, 34 and 31 miRNAs were identified, respectively. The intersection of these miRNAs led to the identification of five key miRNAs (Fig. 9a). Subsequently, 31 lncRNAs targeting these key miRNAs were predicted, resulting in a lncRNA-miRNA-mRNA network comprising 37 nodes and 40 edges. For instance, MRPS11 was linked to ‘hsa-miR-650’-TUG1, and SHMT2 to ‘hsa-miR-346’-GAS5 (Fig. 9b). Additionally, 10 TFs—five targeting MRPS11 and five targeting SHMT2—were predicted, forming a TF-mRNA network with 12 nodes and 10 edges, exemplified by relationships such as REB1_B-MRPS11 and GBF_Q2-SHMT2 (Fig. 9c). Moreover, 15 drugs targeting MRPS11 and 56 drugs targeting SHMT2 were identified, and a biomarkers-drugs network was established, highlighting drugs such as acetaminophen and lycorine that co-target both biomarkers (Fig. 9d). These analyses collectively provide a comprehensive overview of the regulatory networks and potential therapeutic targets associated with MRPS11 and SHMT2.

## Discussion

MDD is a complex and heterogeneous disorder, and no single theory has been proven sufficient to explain its polymorphic nature or identify reliable diagnostic biomarkers [50]. The conventional monoamine hypothesis fails to fully account for the limited efficacy of antidepressants, highlighting the need for additional pathophysiological explanations for MDD. The ES plays a critical role in the network activity that regulates the central nervous system’s response to emotional stimuli [12]. Emerging evidence indicates that the ES significantly influences neurotransmission, as well as the neuroendocrine and neuroimmune systems, all of which contribute to the pathophysiology of depression [51]. Therefore, the complex interactions between the ES and MDD warrant further investigation. This study identified two ES-associated biomarkers in MDD: MRPS11 and SHMT2. These biomarkers may serve as diagnostic and therapeutic tools for MDD, either independently or in combination with others, offering potential for novel approaches in the clinical management of the disorder.

### Biomarker results and RT-qPCR results

MRPS11 is a member of the mitochondrial ribosomal protein (MRPS) family, playing a vital role in mitochondrial ribosome assembly and protein synthesis within the mitochondria [52–54]. Mitochondrial ribosomes differ structurally and functionally from their cytoplasmic counterparts, with the MRPS family being essential for the translation of proteins encoded by mitochondrial DNA [55]. Research on the MRPS family has primarily focused on understanding their roles in mitochondria and exploring their potential as therapeutic targets across various diseases [56]. Several studies have linked MRPS gene expression dynamics to a range of diseases, including cancer, cardiovascular diseases, and neurodegenerative disorders [57]. Notably, MRPS11 has been identified as a potential contributor to cancer pathogenesis. Recent studies have shown that the interaction between LncRNA ZFHX4-AS1 and MRPS11 may be closely associated with the immune microenvironment in ovarian cancer, thereby promoting its progression [58]. Furthermore, MRPS11 expression is significantly downregulated in the peripheral blood of patients with ischemic stroke [59], where it serves as an important biomarker for assessing prognostic risk [60]. In patients with ankylosing spondylitis (AS), MRPS11 expression is also notably reduced, suggesting its involvement in the disease’s pathobiological mechanisms [61]. Additionally, MRPS11 has been implicated in the pathogenesis of chronic obstructive pulmonary disease (COPD) [62].

SHMT2 is a key enzyme in one-carbon metabolism, facilitating the conversion of serine into glycine and a one-carbon unit bound to tetrahydrofolate [63]. Numerous studies have underscored SHMT2’s critical role in maintaining normal methylation profiles, DNA stability, and genetic variation [63]. SHMT2 is essential for cellular function and proliferation [64, 65]. Elevated expression of SHMT2 has been documented in various human cancers, including colorectal, lung, breast, ovarian, and prostate cancers, with higher expression levels correlating with poor prognosis in tumor tissues [63].

SHMT activity is significantly higher in individuals with non-psychotic depression compared to those with psychotic depression [66]. In contrast, individuals with psychosis exhibit reduced SHMT activation relative to healthy controls [67]. Additionally, children with higher frequencies of SHMT C1420T polymorphisms are less likely to develop autism [68]. The mRNA expression of SHMT2 has also been associated with bipolar disorder, further supporting the NMDA receptor (NMDAR) hypothesis of bipolar disorder [69].

Given the role of mitochondrial dysfunction in the pathophysiology of depression, MRPS11 and SHMT2 may represent potential therapeutic targets for this condition [70]. RT-qPCR analysis revealed significantly reduced expression levels of MRPS11 and SHMT2 in samples from individuals with MDD (Fig. 4f). These findings highlight MRPS11 and SHMT2 as biomarkers warranting further investigation. The identification of these biomarkers as potential markers for depression is a significant advancement. However, their validation as reliable diagnostic markers and therapeutic targets requires further comprehensive research. This discovery could enhance our understanding of depression and contribute to the development of novel diagnostic and therapeutic strategies.

### Functional enrichment analysis

GSEA enrichment analysis revealed that MRPS11 and SHMT2 are co-enriched in the “ribosome” pathway, offering preliminary evidence that this pathway may play a significant role in the pathophysiology of MDD, warranting further exploration. Ribosomes, as essential RNA-protein complexes, are responsible for protein synthesis in all cellular contexts. They are crucial for maintaining protein homeostasis and play a central role in regulating gene expression [71]. Ribosomes are also known to be targets of oxidative stress [72]. Consequently, ribosome repair becomes essential for resistance to oxidative stress, as it preserves the integrity of both ribosomes and mRNAs under stress conditions [73]. The involvement of oxidative stress in MDD pathogenesis is well-established. Oxidative stress, driven by free radicals, nonradical molecules, and reactive oxygen and nitrogen species, significantly contributes to the pathophysiology of depression. Products of oxidative stress serve as key indicators for assessing the severity of depression and evaluating the effectiveness of antidepressant treatments [74]. In summary, the potential role of MRPS11 and SHMT2 in depression, through mitochondrial dysfunction and oxidative stress, offers new insights into the pathogenic mechanisms and potential therapeutic strategies for MDD.

### Immunoinfiltration analysis and correlation analysis

Immune infiltration analysis revealed significant disparities in the infiltration of plasmacytoid dendritic cells, memory B cells, immature dendritic cells, and T helper 17 (Th17) cells. Immature dendritic cells, located in peripheral tissues, specialize in antigen capture [75]. Memory B cells, generated in the germinal center after initial infection, are crucial for coordinating the secondary immune response [76]. An earlier bioinformatics study demonstrated a correlation between memory B cells and genes functioning as diagnostic markers for MDD [77]. Plasmacytoid dendritic cells are specialized in the production of type I interferon (IFN-I), which plays a key role in regulating antiviral immune responses [78]. These cells influence both biological and pathological processes, including viral infections and tumors [79]. Recent studies have emphasized their critical role in immune regulation and anti-tumor immunity [80–82].

Th17 cells, a subset of highly pro-inflammatory effector T cells, are vital for the defense against extracellular bacteria and fungi. However, they also contribute to the development of chronic inflammatory and autoimmune diseases [83]. Conditions such as autoimmune encephalitis, inflammatory bowel disease, and respiratory disorders like COPD and asthma have been linked to Th17-mediated inflammatory responses [84]. Th17 cells are also implicated in the neuroinflammation associated with depression, correlating with the severity of the disorder [85]. Animal model studies have revealed the potential involvement of Th17 cells and inflammation in the pathogenesis of depression induced by chronic social stress [86]. This aligns with our finding of increased Th17 cells in the depression group. MDD is characterized by the activation of the immune-inflammatory response system and the compensatory immunoregulatory system (CIRS), alongside a deficiency in T regulatory (Treg) cells [87]. Our findings further support the significant role of immune inflammation in the pathophysiology of depression, thereby advancing the current understanding of this field.

### Biomarker-drug network analysis

Biomarker-drug network analysis revealed that MRPS11 is associated with 15 drugs, while SHMT2 is associated with 56 drugs. Notably, acetaminophen is correlated with both of these biomarkers. Acetaminophen (Paracetamol) is a widely known analgesic and antipyretic agent. However, its pharmacological effects extend beyond pain relief and fever reduction. Extensive research has demonstrated that acetaminophen exerts a range of more complex and diverse pharmacological actions than previously understood. It inhibits prostaglandin synthesis by competitively blocking the peroxidase component of prostaglandin H2 synthase. Furthermore, its metabolite, N-arachidonoyl-phenolamine (AM404), activates transient receptor potential vanilloid subtype 1 (TRPV1) receptors and disrupts CB receptor signaling [88]. AM404 can be detected in cerebrospinal fluid following paracetamol administration. The central analgesic effect of AM404 primarily arises from its ability to increase local concentrations of glutamate, GABA, and endocannabinoids, reducing neural connectivity between the cortex, hypothalamus, amygdala, and periaqueductal gray [89]. Moreover, the involvement of GABA, endocannabinoids, and glutamate in the pathophysiology and psychopharmacology of depression has been increasingly recognized [90].

Acetaminophen possesses anxiolytic effects [91]. Additionally, it exerts antidepressant-like and anticompulsive-like effects, attributed to the enhancement of the serotonergic pathway and the ES [92]. Low-dose acetaminophen, unlike high-dose administration, demonstrates antidepressant-like activity without inducing tolerance or withdrawal symptoms. This effect may also be linked to alterations in the opioid system [93]. Emerging evidence highlights the opioidergic pathway as a crucial factor in the pathophysiology of depression [94]. Furthermore, low-dose acetaminophen treatment has neuroprotective effects [95], potentially improving cognitive dysfunction in depression. These findings suggest that acetaminophen could serve as a valuable therapeutic agent for depression, as supported by our study. Further investigation into acetaminophen’s effects is warranted.

## Conclusion and limitations

This study identified two key biomarkers, MRPS11 and SHMT2, through bioinformatics analysis, and their expression was validated in clinical samples by PCR. Based on these biomarkers, a nomogram model with strong predictive performance was constructed, laying a solid foundation for the clinical prediction of MDD risk. Additionally, functional enrichment, immune infiltration, and regulatory network analyses provided new insights for the diagnosis and treatment of MDD. Based on our research findings and existing literature, we propose the following hypothesis: The dysregulation of the ES, characterized by reduced signaling efficacy, promotes the pathogenesis of MDD [96, 97] by inducing mitochondrial dysfunction and oxidative stress [98, 99]. This cellular energy and redox disorder subsequently led to downregulation of biosynthetic processes, resulting in decreased expression of key mitochondrial genes (such as MRPS11, which is involved in mitochondrial protein translation, and SHMT2, a key enzyme in one-carbon metabolism) [100, 101]. This hypothetical pathway links the observed ES dysregulation with the mitochondrial dysfunction observed in MDD by modulating the expression of these key genes. However, our study has several limitations. First, the ES-RGs identified in this study were limited to only 10 genes selected from the GenBank database, which is a relatively small dataset. Second, the specific mechanisms of action for the MRPS11 and SHMT2 genes have not yet been experimentally validated. Finally, the drug sensitivity was not experimentally verified. In response to the aforementioned limitations, the following work is planned for future research. First, we will expand the database for screening genes to identify more ES-RGs. Second, we will increase the clinical sample size to systematically validate the bioinformatics findings of this study. Finally, we plan to intensify research on regulatory mechanisms, optimize the model, and conduct supplementary studies such as incorporating more features or improving existing feature selection methods. These efforts will help enhance the reliability and clinical translational potential of the research conclusions, provide new insights into the underlying molecular mechanisms and clinical approaches, and contribute to the advancement of targeted and personalized treatment strategies, as well as elucidate the connection between ES and MDD.

## Supplementary Information

Below is the link to the electronic supplementary material.

Additional file 1

Additional file 2

Additional file 3

Additional file 4

Additional file 5

Additional file 6

## Data Availability

All data generated or analyzed during this study are included in this published article. The datasets analysed during the current study are available in the [GEO] repository, [https://www.ncbi.nlm.nih.gov].
